# Synergizing Deep Learning-Enabled Preprocessing and Human–AI Integration for Efficient Automatic Ground Truth Generation

**DOI:** 10.3390/bioengineering11050434

**Published:** 2024-04-28

**Authors:** Christopher Collazo, Ian Vargas, Brendon Cara, Carla J. Weinheimer, Ryan P. Grabau, Dmitry Goldgof, Lawrence Hall, Samuel A. Wickline, Hua Pan

**Affiliations:** 1 College of Engineering, University of South Florida, Tampa, FL 33620, USA; 2 The Heart Institute, College of Medicine, University of South Florida, Tampa, FL 33602, USAbcara98@hotmail.com (B.C.); wickline.sam@gmail.com (S.A.W.); 3 Department of Medicine, Washington University in St. Louis, St. Louis, MO 63110, USA; 4 Department of Pathology & Immunology, Washington University in St. Louis, St. Louis, MO 63110, USA; 5 Department of Biomedical Engineering, Washington University in St. Louis, St. Louis, MO 63130, USA

**Keywords:** machine learning, active deep learning, ground truth, convolutional neural network, deep learning-based preprocessing

## Abstract

The progress of incorporating deep learning in the field of medical image interpretation has been greatly hindered due to the tremendous cost and time associated with generating ground truth for supervised machine learning, alongside concerns about the inconsistent quality of images acquired. Active learning offers a potential solution to these problems of expanding dataset ground truth by algorithmically choosing the most informative samples for ground truth labeling. Still, this effort incurs the costs of human labeling, which needs minimization. Furthermore, automatic labeling approaches employing active learning often exhibit overfitting tendencies while selecting samples closely aligned with the training set distribution and excluding out-of-distribution samples, which could potentially improve the model’s effectiveness. We propose that the majority of out-of-distribution instances can be attributed to inconsistent cross images. Since the FDA approved the first whole-slide image system for medical diagnosis in 2017, whole-slide images have provided enriched critical information to advance the field of automated histopathology. Here, we exemplify the benefits of a novel deep learning strategy that utilizes high-resolution whole-slide microscopic images. We quantitatively assess and visually highlight the inconsistencies within the whole-slide image dataset employed in this study. Accordingly, we introduce a deep learning-based preprocessing algorithm designed to normalize unknown samples to the training set distribution, effectively mitigating the overfitting issue. Consequently, our approach significantly increases the amount of automatic region-of-interest ground truth labeling on high-resolution whole-slide images using active deep learning. We accept 92% of the automatic labels generated for our unlabeled data cohort, expanding the labeled dataset by 845%. Additionally, we demonstrate expert time savings of 96% relative to manual expert ground-truth labeling.

## 1. Introduction

Deep learning and convolutional neural networks have proven to be highly effective and useful tools in medical image analysis, including MRI, CT, PET, and histopathology images [1,2,3,4,5,6,7,8]. Even so, expert labeling of medical image datasets to generate ground truth for training deep learning algorithms is still an ongoing challenge to the adoption of artificial intelligence in medical practice [7]. In an ideal world, all possible medical images would be curated, labeled, collected, and available at any time for anyone to train universally robust machine learning algorithms, and significant efforts have been devoted to generating medical image datasets with ground truth for research communities [9]. However, development of such datasets is challenging due to limitations on resources and time. The cost of many medical expert hours spent hand-labeling data for deep learning training sets is often so high that crowdsourcing has been employed as an alternative [10,11,12,13]. Besides the potential inaccuracy of ground truth generated through crowdsourcing [10], the risk of exposing protected patient information also exists [14]. Laws and standards differ between localities, regions, and nations, with the US stringently gating the public release of medical information due to privacy concerns [15,16]. In other studies involving deep learning, images are substantially downsized to reduce the need for computational and expert resources [17,18].

Medical images, including pathological microscopic whole-slide images, are often collected in isolation or as a cooperative effort between institutions. When images are acquired, the imaging device or microscopy, curation, and labeling are typically performed at different times, by different individuals, using different equipment, and with different specimens or different patients. Because of this, image features can be inconsistent across a dataset, as we demonstrate quantitatively in this paper. Accordingly, automated methods are often sensitive to these variabilities, producing questionable results [19,20,21]. Therefore, the selective removal of ‘poor-quality’ images has been proposed [22], offering benefits in developing deep learning for artificial intelligence (AI)-assisted medical image diagnosis in specific medical settings. On the other hand, some research efforts focus on developing preprocessing algorithms to include all images for AI-based diagnosis [23,24,25]. Because experienced pathologists/radiologists typically look beyond the ‘poor-quality’ aspects and identify valuable features in microscopic images to provide accurate diagnostic evaluations, the present work seeks to integrate human and machine intelligence. We aim to automatically generate additional ground truth with an active deep learning (ADL) and semantic preprocessing methodology, which could promote AI-assisted medical image analyses capable of including ‘poor-quality’ images. Our approach integrates deep learning with preprocessing, establishing a positive feedback loop between image preprocessing and a downstream CNN model. Additionally, our method combines deep learning-enabled preprocessing with human–AI collaboration to streamline the process of generating ground truth annotations. By leveraging deep learning for preprocessing tasks to normalize ‘poor-quality’ images toward high-quality ones and integrating AI algorithms with significantly reduced human expertise effort and time, our approach achieves unprecedented efficiency and accuracy in AI-assisted ground truth generation. This synergistic approach not only reduces the time and effort required for annotation tasks but also enhances the quality and reliability of the generated annotations.

Active learning has been a highly promising potential solution to generating ground truth with minimized human input. In the active learning domain, various researchers have contributed valuable insights to enhance sample selection and improve model generalization. Cohn et al. introduced a theory on sample selection and generalization, emphasizing the effectiveness of formal sample selection methods in scenarios where labeling incurs significant expenses [26]. Li et al. proposed a harm-reduction strategy for unlabeled samples in semi-supervised learning with Support Vector Machines (SVMs), selecting those with the lowest risk factor to optimize performance [27]. Raczkowska et al. demonstrated the utility of variational dropout in creating an ensemble or committee of models, useful for measuring certainty and informing sample selection decisions [28]. Sourati et al. innovatively derived Fisher information from deep learning model parameters to supply a measure of certainty based on an approximate transformation of these parameters [29]. DeVries et al. explored training a secondary model to evaluate segmentation model uncertainty, assisting experts in identifying spatial areas in the images requiring additional attention [30]. Huang et al. showcased the effectiveness of saving snapshots of a model during training, creating an ensemble that leverages the model’s shifts around the error surface [31]. While most variants of active learning work by selecting the most useful samples for the expert to label the region of interest (ROI) according to a carefully considered uncertainty metric, this still requires manual ground-truth generation by hand, and is not ideal due to the required time and cost [32].

Alahmari et al. from our group introduced an iterative improvement method involving expert evaluation of unknown data, incorporation into the training set, and retraining for model refinement [33]. Building upon Huang et al.’s approach, a snapshot ensemble, as demonstrated by Alahmari et al., utilizes voting to produce confidence values, facilitating the identification of the best samples and reducing expert workload [34]. Alahmari et al. pioneered a novel approach called “active deep learning” (ADL), whereby a snapshot ensemble votes to automatically label samples, submitting the highest-confidence (highest vote proportion) samples for approval by the expert [34]. This minimizes the expert’s input to a simple “accept” or “reject” decision for each sample. Unfortunately, owing to the aforementioned imaging inconsistency issues, ADL manifests a pattern of diminishing returns, accepting only a subset of the active set for inclusion into the dataset (55% after five iterations).

We hypothesize that the confidence metric of ADL is selecting the subset of samples that most closely resemble the training data distribution while rejecting out-of-distribution samples, which implies a classic overfitting scenario. We believe that inconsistent microscopic images induce the bulk of this overfitting. By quantifying how both the active set and the universe of potential unknown data differ from the training set, a preprocessing algorithm can be devised that normalizes new samples to the training set’s distribution, thereby increasing active deep learning confidence scores and sample acceptance rates. This improvement could further reduce human effort by integrating artificial intelligence and human intelligence.

Here, we present a fusion of Alahmari et al.’s snapshot ensemble ADL with a novel Bootstrapped Semantic Preprocessing (BSP) on input images that remedies the overfitting issue. In this context, semantic preprocessing (SP) refers to a preprocessing method wherein adjustments can be made more consistently than with methods based on local or histogram metrics. We do this by obtaining a semantic metric relevant to the dataset distribution from areas of interest in the deep learning model’s prediction. A typical semantic metric would be the mean pixel value of each region. These regions and metrics, which are unknown before prediction and obtained by analyzing the prediction, can be used to retroactively make preprocessing adjustments via a bootstrapping process. We also boost the performance of this new algorithm by augmenting it with a gradient descent process using multiple types of image processing techniques in series.

Our semantic preprocessing (SP) methodology considers class-based image features, derives a simple metric from these features (typically the mean brightness of each class area in this case), and then adjusts the image according to this feature-aware or semantic metric. By repeating this process iteratively for unknown, unlabeled data using the bootstrapping technique [35], an ADL snapshot ensemble can normalize unknown samples to the training set distribution, increasing prediction quality, mitigating overfitting issues, and improving the likelihood that evaluated samples will be accepted by the human expert. We demonstrate these gains on full-resolution whole-slide images with no downsizing or loss of detail with the use of patch-wise interpolation. This SP methodology also is suitable for application to diverse medical imaging data beyond simple histology.

## 2. Materials and Methods

### 2.1. Materials

#### 2.1.1. Microscopy Imaging

To demonstrate our method, we used fluorescence microscopy images of mouse hearts to analyze the extent of ischemic tissue damage following experimental acute myocardial infarction (AMI). All animal experiments were performed in compliance with guidelines and protocols approved by the Division of Comparative Medicine at Washington University in St. Louis. The animal protocol is subjected to annual review and approval by The Animal Studies Committee of Washington University in St. Louis. The collection process was as follows:

Twenty C57BL6 mice underwent left anterior descending (LAD) coronary artery ligation for 90 min, followed by reperfusion for 24 h, before euthanasia. After the hearts were excised and perfused with saline retrograde through the aortic root, the LAD coronary artery was reoccluded, and 50 µL of Lycopersicon Esculentum (Tomato) Lectin (LEL, TL), DyLight^®^ 488 (Cat#: DL-1174-1, Vector Laboratories, Burlingame, CA, USA) (hereafter referred to as lectin-488) in 1 mL saline was perfused to label all blood vessels located in the nonischemic region. The hearts were then cut into five sections, embedded in O.C.T. (Cat#: 23-730-571, FisherSci, Pittsburgh, PA, USA), and sectioned at 8 µm for fluorescence imaging. Before imaging, heart sections were fixed and washed, before being mounted with VECTASHIELD^®^ Antifade Mounting Medium with DAPI (Cat#: H-1200-10, Vector Laboratories, Burlingame, CA, USA) for imaging by using an Olympus fluorescence microscope. Images were acquired at selected wavelengths matched to the specific fluorophores. A total of 100 sections (two images per section) were obtained for (1) nuclear stains, which defined the region of tissue sections within the acquired high-resolution images, and (2) lectin-488, which defined the myocardial regions that had maintained normal blood flow throughout the experimental procedures. Image sets in Cohort 3 also contained dystrophin stainings that were registered with an Alexa Fluor 594 secondary antibody, which was noted upon subsequent analysis to have introduced slight bleed-through into the lectin-488 channel.

#### 2.1.2. Dataset Arrangement

*(1) Cohorts and Samples:* The 100 sample sections collected are split between three cohorts:

- Cohort 1: 11 sections from 11 mice, with expert ground truth.
- Cohort 2: 45 sections from 9 additional mice.
- Cohort 3: 44 sections from the same 9 mice as Cohort 2.

Cohort 1, having ground truth, is split into the initial training, validation, and cross-validation test sections. Cohorts 2 and 3, having no ground truth, comprise the active set, from which sections were integrated into the training set, as active learning generates approved ground truth ROI labels. Each section image, taken at 162.5 nm/pixel of resolution, is downscaled by a factor of 0.3, resulting in a resolution of 541.67 nm/pixel and roughly 10,000 pixels per side. However, these images are high resolution and too large to be evaluated as a whole by a neural network of a reasonable size for computation. For prediction, these section images are segmented into a grid of 512 × 512-pixel patches, which serve as the input samples to the neural network, as denoted by the sample counts in Table 1.

The large number of sections in the unlabeled cohorts provided an opportunity to expand our available ground truth by a potential multiple of 5 in a perfect scenario. However, even without this perfect result, we could label an amount of data much larger than Cohort 1. In addition, these cohorts display significant inter-cohort and intra-cohort variability. We analyzed this variability in the *Dataset Inconsistency* (Section 2.1.2 (4)) and performed a more in-depth analysis, as well as testing of our semantic preprocessing method’s effectiveness at normalizing this variability, as presented in Supplementary Discussion S1.

**Figure 1:**
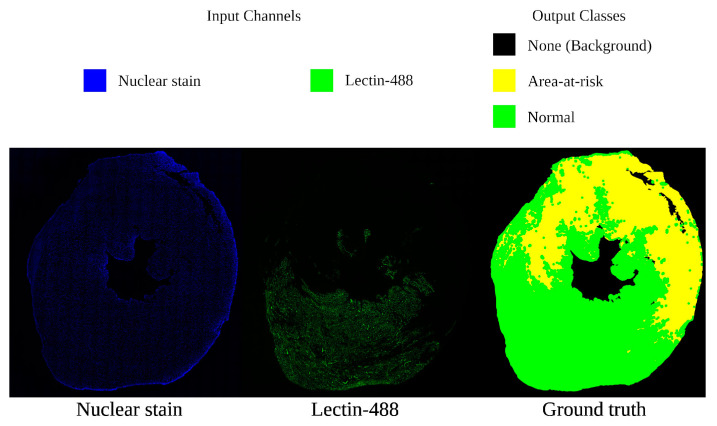
A sample section from the dataset, with a legend for interpreting false coloration. On the left is the nuclear stain channel identifying total tissue area; in the center is the lectin-488 channel identifying blood reperfusion; on the right is the ground truth map, with green representing normal tissue, yellow as area-at-risk (AAR), and black as the non-tissue external region.

*(2) Input and Output:* Each section supplies two images (nuclear stain and lectin-488) that served as the inputs for our neural network; for the input channels, nuclear stain is color-coded as blue, and lectin-488 is color-coded as green, as shown in Figure 1.

The goal of our network is to segment the samples according to whether any given region of pixels corresponds to one of three data categories: (1) the background of the slide outside of the tissue section, (2) the ischemic area-at-risk (AAR) of cell death that is not perfused with blood (or, the lectin negative areas), and (3) the nonischemic area of myocardium with normal blood perfusion (lectin positive areas). Thus, our neural network will output three channels: “None” (background, coded black); “risk” (coded yellow); and “normal” (coded green). These names and colors will be used whenever we visualize a ground truth or output ROI segmentation map and can be referenced in Figure 1.

All input images were converted to 8-bit pixel depth, yielding grayscale intensity values from 0 to 255, 0 being black, and 255 being white; the colors indicated in Figure 1 are false colors for clarity of interpretation. Note that for the remainder of this paper, “brightness”, “intensity”, and “pixel value” are used interchangeably to refer to this 0-to-255 value. Considering the patch-grid split previously covered in *Cohorts and Samples* (Section 2.1.2 (1)), we can now consider our individual neural network inputs and outputs. For inputs, when we combine the two image channels, we have a 512 × 512 × 2 patch. As output, we have a 512 × 512 × 3 confidence map patch where each pixel’s values indicate the model’s proportional confidence in each class at that pixel. For final segmentation, the highest proportion is chosen as that pixel’s class. During prediction, the output patches of the neural network are stitched together to form a whole section, as explained in depth below.

*(3) Ground Truth:* Expert ground truth was generated for Cohort 1’s 11 sections. A full ROI segmentation map containing none, risk, and normal regions was “hand-painted” in GIMP (GNU Image Manipulation Program) [36], such that for each tissue class, every pixel in the entire section image is assigned to one of these classes. The lectin-488 signal closely matches normal vascular structures; however, “normal” healthy tissue can fall as many as 26 µm outside of the lectin-488 vascular signal area, using the assumption that the nucleus is in the center of the cells and cell walls fall on average 26 µm away from the nucleus. This margin is taken into account when constructing the ground truth class map. Figure 1 gives an example of one of these 11 sections’ input channels and ground truth segmentation map.

**Figure 2:**
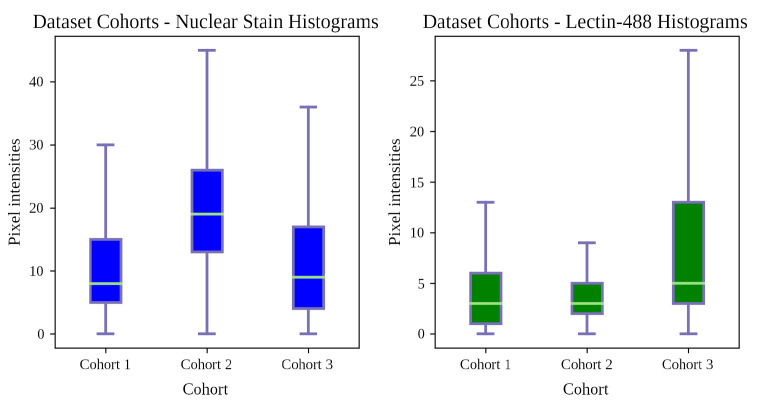
Histograms showing distributions for dataset cohorts. Cohort 1 is the training and cross-validation data, while Cohorts 2 and 3 are the active sets.

*(4) Dataset Inconsistency*: The distributions for each cohort’s channels may vary considerably. Figure 2 shows how Cohort 2’s nuclear stain channel is exceptionally bright, with most of it corresponding to Cohort 1’s upper quartile. Cohort 3’s lectin-488 channel skews bright, with its upper quartile exceeding Cohort 1’s maximum. Despite adhering to the defined microscopic acquisition parameters, deviations from the training set (Cohort 1) distribution, e.g., signal intensity, were observed. Such deviations should be anticipated and appropriately addressed. The aim of SP is to normalize these cohorts robustly for effective deep learning. A more thorough analysis of dataset inconsistency is presented in the Supplementary Discussion S2.

#### 2.1.3. Deep Learning Model

**Figure 3:**
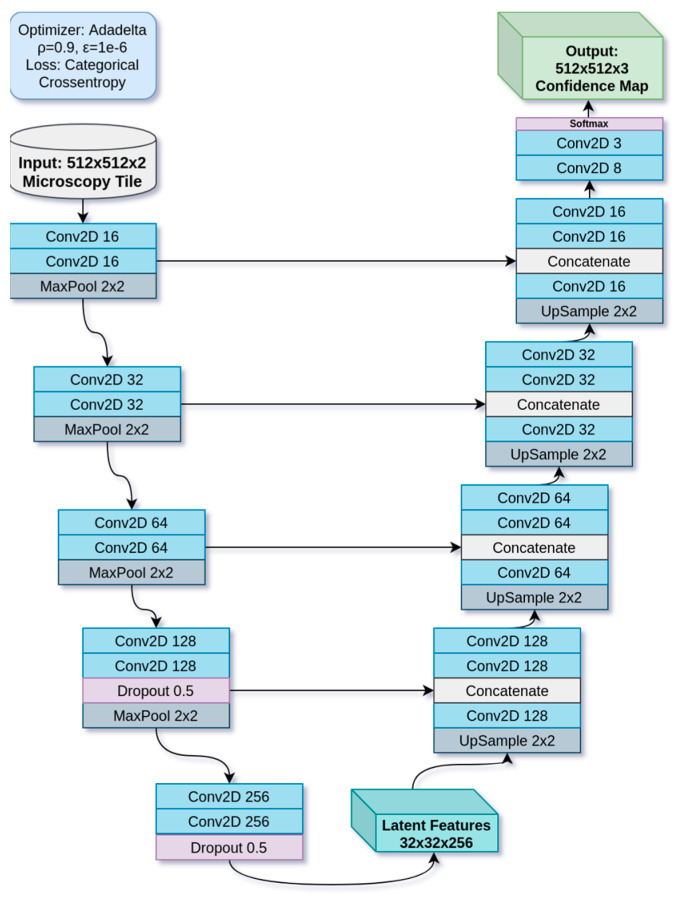
A U-Net-based model used in this experiment.

For all experiments in this paper, we will be using a deep neural network based on the UNet encoder–decoder architecture [36], but downscaled to approximately 2 million parameters. UNet is a commonly used architecture for image segmentation problems [37], especially in histopathology, because its residual connections forward fine-grained details from the encode path to the decode path for fine-grain segmentation. Our version is built using PyTorch [38] with convolutional layers [39] to extract information about texture and shape. Its architecture is laid out in Figure 3. The UNet is configured to translate a two-channel fluorescence patch input into a three-class segmentation patch. Thus, the two fluorescence channels are fed as input together, while three confidence values are emitted, one for each class, and the highest is chosen to produce the segmentation map. The input patches, with their corresponding ground truth segmentation patches, are used to train the network through backpropagation. All inputs are normalized to [0.0, 1.0] floating-point. The model performs gradient descent [40] using the Adadelta algorithm [41], with = 0.9 (decay rate), = 1 × 10^−6^ (numerical stability term), and gradient norm clipping of 1.0 to prevent an exploding gradient. Loss is calculated using categorical cross-entropy [40]. Accuracy is measured using the Dice score as shown in Equation (1), where A and B denote images being compared.

(1) $$Dice=\frac{2\times \left|A\cap B\right|}{\left|A\right|+\left|B\right|}$$

### 2.2. Methods

In this paper, we present a preprocessing technique and two novel methodologies using this technique for automatically labeling histopathology images using ADL, and perform experiments to demonstrate their effectiveness. First, we present the semantic preprocessing (SP) technique, which derives a feature-dependent metric from a prediction map, and then adjusts the image to match a target metric. We then present a fusion of SP with an ADL approach, whereby a snapshot ensemble voting process is augmented with a bootstrapped version of semantic preprocessing (BSP), increasing the production of usable ground truths from ADL significantly. From there, we improve upon BSP with a gradient descent algorithm (GDBSP) that synthesizes multiple image processing algorithms together in series.

#### 2.2.1. Semantic Preprocessing (SP)

**Figure 4:**
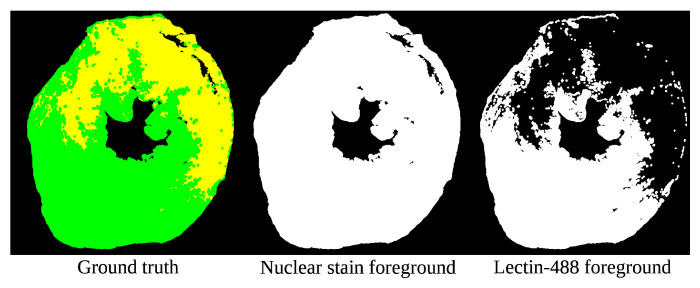
Foreground/background ROI segmentation for a sample section, for both input channels. Each of these will be used to adjust their corresponding microscopy image independently with semantic preprocessing. In the Ground truth, green + yellow indicates nuclear stain, while green represents the Lectin-488 signal.

**Figure 5:**
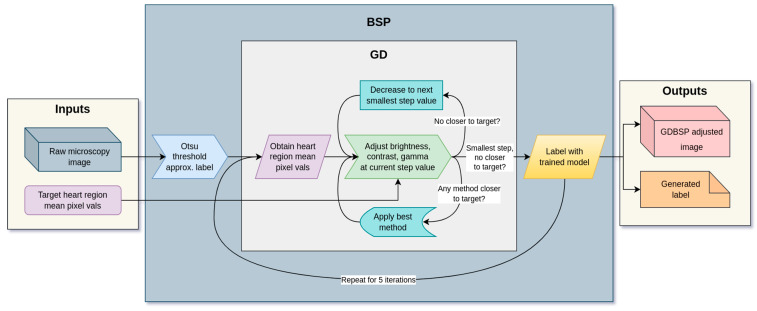
Diagram outlining bootstrapping iteration model and gradient descent process for evaluating unknown data (BSP and GDBSP). Note that the GD algorithm is a subprocess of the overall BSP algorithm. Simple BSP substitutes the GD portion for a simple gamma-based distance method.

Semantic preprocessing (SP) is the novel core of our image-processing approach. It involves two important steps: extracting a metric from an image, and adjusting the image such that the metric moves closer to a target value. In our experiment, we implement a basic variant of this methodology. Each heart section consists of two images (examples shown in Figure 4), each of which needs accompanying binary segmentation information: the nuclear stain image is associated with “none” vs. “non-none” regions, whereas the lectin-488 image displays “normal” vs. “not-normal” regions. This segmentation can be obtained either by approximation or evaluation; in this case, we used Otsu thresholding as an approximation. We extracted the mean pixel value of the two regions (thus, the mean background brightness and the mean foreground brightness). We treated these two values as a two-dimensional coordinate; we extracted the same from a known high-quality section, and used that as a target. We then iteratively adjusted the gamma of the image in 5% steps up or down depending on which moved us closer to the target. When either adjustment moved us further away, we stopped. We then obtained our resulting adjusted image. The detailed process for executing this distance-minimization brightness adjustment is depicted in Figure 5.

#### 2.2.2. Bootstrapped Semantic Preprocessing (BSP)

Bootstrapped semantic preprocessing (BSP) takes advantage of the statistical phenomenon of bootstrapping [35]: When we take an SP-adjusted image and re-sample it with a trained model, then repeat for some number of iterations, we asymptotically converge toward a stable adjusted image and label that is almost always much higher quality than the Otsu approximation or initial model evaluations. Thus, we apply SP in 5 iterations: First, on the Otsu approximated segmentation. Then, each SP-adjusted image is fed to the deep learning model to produce a new segmentation map (label) that is used to run SP. We then converge on a high-quality segmentation and a “best”-adjusted version of the input images. To produce a quality ground truth data label when starting with no ground truth or only an approximation, we can predict the samples iteratively, converging toward a quality label. For this dataset, analyses and hypotheses concerning our data were formulated to inform the bootstrapping process.

**Figure 6:**
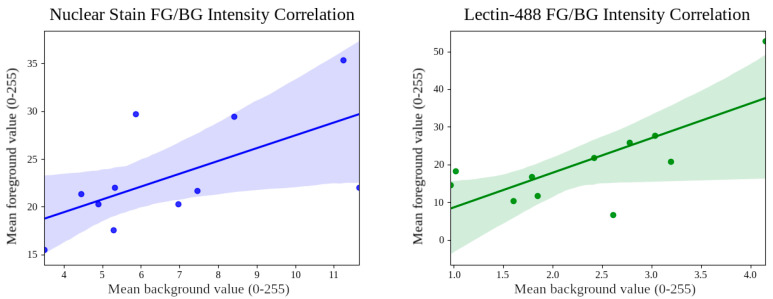
Correlation plots for the Cohort 1 nuclear stain and lectin-488 channel average class area pixel values. In both scenarios, the average pixel value for each class area is positively correlated; thus, they are being affected similarly by global microscopy session conditions. Hyperbolae are 95% confidence intervals. For nuclear stain (blue), correlation coefficient is 0.61, r-squared is 0.3738, and *p*-value is 0.0456. For lectin-488 (green), correlation coefficient is 0.72, r-squared is 0.5198, and *p*-value is 0.0123.

The BSP method is, as we will soon show, adaptable to any combination of adjustment algorithms desired, but for this dataset and a simple BSP implementation, we selected only gamma adjustment. This is because the foreground and background average intensities for each of our two input channels are positively correlated, as shown in Figure 6. We observed that for nuclear stain (blue), the correlation coefficient is 0.61, r-squared is 0.3738, and *p*-value is 0.0456. For lectin-488 (green), the correlation coefficient is 0.72, r-squared is 0.5198, and *p*-value is 0.0123. This suggests that exposure is the most vulnerable property of fluorescence imaging, dependent on variables including biomarker density, exposure time, whole-slide microscopy stitching algorithm consistency, and more. By adjusting the exposure by whole-image gamma adjustment, we can exploit those correlated class intensities to adjust the brightness of the whole image and reach an intensity target. Because foreground and background pixel values are correlated, we hypothesized that a somewhat inaccurate ROI segmentation map with contracted or expanded borders but majority coverage will exhibit class average pixel values correlated with those of an accurate segmentation map. Therefore, using a binary (two-class) Otsu thresholding [42] on both foreground and background to generate preliminary approximate segmentation maps, we can adjust the raw input images, and then repeat steps of trained model prediction followed by SP using the resulting segmentation maps, generating better segmentation maps each time through a bootstrapping process. Note that at each step, adjustments are applied to the original raw images in the data collection cohort, not to the adjusted images from the previous step, to ensure that any erroneous adjustments do not destroy information needed in future steps. Through this process, the model effectively normalizes unknown data to a state more consistent with the training set distribution. The process is flow-graphed in Figure 5.

#### 2.2.3. Gradient Descent BSP (GDBSP)

Gradient descent BSP (GDBSP), building upon and adding adjustments to BSP, steps down a semantic gradient much more deliberately. We consider both the mean and standard deviation of each class area’s pixel values as the semantic metrics, and probe the gradient toward the target image metric using a combination of contrast, brightness, and gamma. Thus, each additional class in the image adds two dimensions to the semantic space. In our experiment, where binary segmented images were adjusted independently, we navigated a four-dimensional gradient (two mean dimensions, two standard deviation dimensions).

First, as with BSP, we obtained our approximate class map, starting with an Otsu thresholding. Then, we extracted our metrics (mean and standard deviation of each class area). We adjusted the image by each metric using a large step: contrast, brightness, and gamma each by 10%. We measured which method gets us closest to the target image’s class area means and standard deviation. Then, we applied that change and continued. If we could no longer close the distance with a 10% step, we reduced the step by 1% at a time (initially stepping down to 9%). Eventually, the image was picked from minute adjustments of contrast, brightness, or gamma 1% at a time until no change could bring us closer to the target, at which point we stopped. Then, again as with BSP, we used our trained ensemble to evaluate and vote on a new segmentation map with the adjusted image. We repeated this for five iterations. The full process is shown in Figure 5.

#### 2.2.4. Active Deep Learning (ADL)

Active learning is a process by which machine learning algorithms of all types can grow their training set and accuracy by predicting unlabeled samples, and then calculate the quality score that could be used to determine the most useful samples to submit for expert labeling [32]. However, this process can still leave the human expert with a significant workload.

Alahmari et al. proposed an automatic labeling variant of active learning called “active deep learning” (ADL), targeting deep learning, convolutional neural networks, and the minimization of the workload of the expert [34]. Using snapshots of the same model over the course of its training, thereby capturing differences in predictions dependent on each snapshot’s position upon the error gradient surface, an ensemble of these snapshots can be used to vote on predictions by choosing the plurality result at each pixel, then calculating confidence from the agreement on that vote. Expert workload is minimized by only submitting the highest-confidence predictions to the expert, and only for approval or rejection for inclusion in the training set, rather than manual labeling. Applied iteratively, this ADL process can produce additional samples for inclusion into the training set after each iteration.

However, this process yields diminishing returns on each iteration [34]. Many samples will end up discarded. We hypothesize that Alahmari’s ensemble confidence vote is overfitting for samples that more closely resemble the training set distribution, in terms of texture, feature distribution, brightness levels, etc. When microscopic images are inconsistent, out-of-distribution samples will produce the lowest quality scores and ensemble confidence votes. After each iteration, the ensemble will deepen its preference for the feature properties it is already choosing due to their increasing frequency in the training set.

Here, we augment Alahmari’s iterative ensemble ADL process by deeply integrating BSP, thereby promoting a reversal of this diminishing return trend by normalizing out-of-distribution samples to the distribution of the training set, leading to the acceptance of more samples into the training set.

*(1) Process:* Our modification of the Alahmari et al. active deep learning process yields the following procedure:

(1) Train the model on the core training data (already preprocessed) for 110 epochs; save snapshots at epochs 90, 100, and 110, creating an ensemble of 3 models

$$M=\left\{M_{90},M_{100},M_{110}\right\}.$$

(2) Test the ensemble against test data for cross-validation.

(3) Use the final, most-trained snapshot M110 to preprocess and predict sections in the active set Z.

(4) For each section in the active set, for the two remaining snapshots in the ensemble $\left\{M_{90},\;M_{100}\right\}$, predict on the given section using the adjusted inputs generated by the epoch 110 model M110.

(5) Calculate a per-pixel vote by the ensemble for the given section. The plurality class for each pixel represents the ensemble’s segmentation of the section Z_ensemble_. The odd number of models prevents ties in cases of even class counts. The proportion of votes for the winner at each pixel comprises the confidence map Z_conf_. The confidence f is the computed average of Z_conf_ across the whole section.

(6) The expert decides on a confidence threshold f_thresh_. This threshold is chosen to yield a number of sections that can be evaluated in reasonable time; for 1 h of work, 12 sections above f_thresh_ is the target. These thresholds are chosen for each iteration at the end of training, and so will change over time.

(7) Leave sections with f < f_thresh_ in the active set. Sections with f ≥ f_thresh_ are evaluated by the expert to accept or reject for inclusion in the training set.

(8) Begin again at Step 1, where the training set is expanded with accepted sections. Adjusted images are the inputs, and plurality-voted ensemble segmentations Z_ensemble_ are the ground truths.

**Figure 7:**
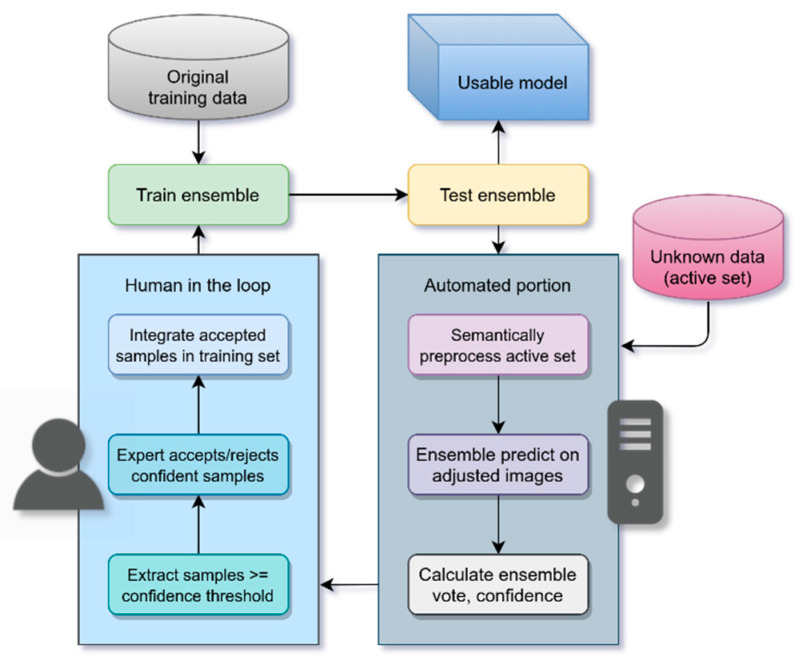
Our modified active deep learning (ADL) process, derived from Alahmari et al. [34].

These steps are depicted in Figure 7. We chose 110 epochs as a carry-over from previous algorithmic experimentation because 100 epochs were found to be computationally feasible while maximizing training loss stability. An extra 10 epochs were added to create a tiebreaker snapshot in a snapshotting strategy for every 10 epochs starting from epoch 10. This was reduced to a last-three snapshots strategy given our tailing loss stability region and in view of the prior findings of Loschilov et al. [43].

An expert hand-picked section is the brightness target for histogram matching, BSP, and GDBSP. By running BSP and GDBSP using the final snapshot, we can obtain the highest quality result using the most-trained snapshot; we then calculate the consensus of the remaining snapshots. Using one of the two earlier snapshots to preprocess the section has a higher probability of a lower-quality adjustment. The handpicked confidence threshold ensures that the work performed by the expert is both doable in a reasonable time frame (~1 h) and that only the highest-quality samples are selected, negating the need for the expert to re-evaluate the entire active set each iteration (as in the Alahmari’s ADL method) [34]. A minimum threshold of 90% is used to filter methods with low-confidence (and thus unstable) ensembles, ensuring that time and work are not wasted.

Because the specific application of ADL in this experiment is image segmentation, and each pixel is subject to classification, we are dealing with input and output parameters numbering per section on the order of 100,000,000. It is inevitable that some noise will be introduced, such as with dust particles, smears, and the like. Thus, quick edits were admitted during expert evaluation that are reasonable within the timespan of analyzing a section for acceptance or rejection; actions such as erasing noise particles or bucket filling areas that are clearly misclassified. For samples that are accepted, combined evaluation and edits should take a mean of 5 min per whole section, whereas, for rejected samples, an analysis and rejection decision takes 1 min on average. If fine-detail manual edits might be required on longer timescales similar to the several hours required for manual ground truth generation, the section is rejected. This maintains the spirit of Alahmari et al.’s minimization of the expert’s labor, while allowing us to evaluate images with orders of magnitude higher resolution.

*(2) Competing Preprocessing Techniques:*

Simpler histogram-based methods are common preprocessing techniques, and we compare our preprocessing algorithms to four of these to benchmark their performance. These techniques are Difference of Gaussians, histogram equalization, adaptive histogram equalization, and histogram matching. The original raw microscopy images with no preprocessing were used as a control.

Difference of Gaussians (DoG) subtracts two versions of an image: one with a higher Gaussian blur, and one with a lower (higher and lower referring to the standard deviation of the Gaussian convolution operator). The effect is a band-pass filter that preserves a band center (between the two blur levels) while attenuating signals outside that center.

Histogram equalization (HE) works by obtaining the cumulative distribution of the image’s pixels, and redistributing the most common values, mapping values in the source image to a new value that ensures a uniform probability distribution function and a linear cumulative distribution function. By this “flattening” and linearizing, histogram equalization acts as a special form of the more general “histogram specification” algorithm targeting the uniform distribution. Note that this method does not use semantic features: features such as class areas and texture affect it indirectly, and it does not consider the dataset distribution. However, for human analysis, the inconsistent contrast is often considered a feature rather than a bug, helping experts to glean details they might otherwise miss.

Adaptive histogram equalization (AHE) applies histogram equalization as a sliding window operation, applying its transformation function to the center pixel in each window. In addition, to prevent over-amplification of near-constant regions, the cumulative distribution function is clipped before applying the transformation to limit contrast.

Histogram matching (HM) is another case of histogram specification where the target histogram is not a uniform distribution but instead another image’s histogram. Thus, the pixel remappings are chosen to match the distribution curves of the target. This is also not a semantic method, functioning without regard for features of class areas; an adjustment that works for a two-class image with a small foreground may yield odd contrast changes for images with large foregrounds.

#### 2.2.5. Experiments

Two experiments were performed to evaluate our BSP active learning methodology: a verification experiment on only Cohort 1, utilizing expert-made ground truths to confirm that BSP and active learning are indeed improving our ensemble; and a large real-world unknown-data experiment, using Cohorts 2 and 3 as the active set, to demonstrate the speed and volume of data labeling possible using our methodology.

*(1) Cohort 1 Experiment*: An initial experiment using only Cohort 1 was performed to verify that preprocessing and dataset growth by active learning was in fact improving performance. Because Cohort 1 has expert-created ground truths, accuracy could be measured on both test data and the active set being evaluated.

The 11 Cohort 1 sections were split into two subsets, with six sections for training, and five as the active set. Six-fold leave-one-out cross-validation was performed on the training portion using a 4/1/1 train/validation/test split for each fold; each section acted as the test section for one fold.

Each iteration for each preprocessing method was conducted as follows:

(1) The six-fold ensembles are trained; any accepted sections are included in the training set for all folds.
(2) Each ensemble is tested for cross-validation.
(3) The least overfit fold (lowest test Dice) ensemble preprocesses active set sections using the given method, predicts, and then votes on, the final composite prediction maps.
(4) With ground truths as the reference, record Dice accuracy, confidence per-section confidence, and mean confidence.
(5) A human expert evaluates active set sections that reach at least 97% confidence, deciding to accept or reject the training set.
(6) Accepted samples are integrated into the training set for the next iteration.

The ”least overfit” fold for each iteration is used to pick the ensemble with the lowest likelihood of settling in untenable local minima, using model parameters with the greatest probability for improvement. An overfit model has a high probability of producing high-confidence votes on poor-quality labels, so our approach avoids this scenario by picking samples that produce high agreement even from a lower-fit model.

While metrics during training and validation are tracked using only the 512 × 512 input and output patches, for testing, we stitch together all the predicted patches of the test section to reconstruct the whole section. However, the model creates some amount of noise at the patch edges due to the lack of context past those edges, leaving a visible grid pattern in the stitching. To combat this, we only take the 256 × 256 center of the output patch, discarding a 128-pixel buffer zone on all sides as trim, and slide our 512 × 512 window a smaller distance of 256 pixels at a time. This method of patch-wise ” interpolation” results in a slightly longer prediction time in exchange for a very consistent full-section ROI segmentation map. Final performance metrics were defined by comparing these full-section predictions of the test section to the test section’s ground truth.

We also record how many sample patches are both 97% confident and accepted by the expert each iteration. This 97% threshold was chosen in earlier experiments as the median confidence of BSP; it often outperformed other methods so thoroughly that the majority of the active set included each iteration using lower thresholds. Thus, for the Cohort 1 experiment, the threshold needs to be strict to identify a difference. Note that the expert evaluates and accepts whole sections such that these figures reflect the sample counts of the combined sections.

*(2) Cohorts 2 and 3 Experiment*: To test how robust our BSP method is in a real-world active learning scenario, we performed three iterations (when yields allowed) of active learning for the four preprocessing methods of raw unaltered images (the control), histogram equalization, histogram matching targeting the test section, BSP, and GDBSP. Three iterations were chosen to save on computing cost and time, as the multi-fold structure of the experiment is extremely computationally intensive. To confirm effectiveness after these comparisons, the best method, as measured at that third iteration (GDBSP), was further iterated until it yielded no further samples, to serve as an example of its performance through an entire dataset.

Each preprocessing method was evaluated in four folds, with the data generated once randomly and used for all methods in all iterations. The Cohort 1 sections acted as the base dataset, with every Cohort 1 section in one fold’s test set, using the following train/validation/test splits: Folds A, B, and C at 6/2/3, fold D at 7/2/2. The unlabeled data used for the active set Z consists of Cohorts 2 and 3; when samples are accepted, all accepted samples are added to the training set for all four folds in subsequent iterations.

Each iteration for each preprocessing method is performed as follows:

(7) The four-fold ensembles are trained; each training set is expanded with any accepted sections.
(8) Each ensemble is tested for cross-validation.
(9) The least overfit (lowest test Dice) fold ensemble is used to preprocess active set sections using the given method, predicts, and then votes on, the final composite prediction maps.
(10) Record per-section confidence and mean confidence on the active set.
(11) A human expert chooses a confidence threshold that produces roughly one hour of work, or approximately 12 sections. Sections above the threshold are evaluated for acceptance or rejection.
(12) Accepted samples are integrated into the training set for the next iteration.

Statistical significance from iteration to iteration is calculated using a T-test for related samples, counting each whole section as a sample and its Dice score as the sample statistic.

The experiment was also attempted using a cyclic learning rate, in the form of cosine annealing with warm restarts, using SGD, two experiments with peak learning rates of 0.1 and 0.8, over 550 epochs with a restart period of 50 epochs and period multiple of 1 [31,43]. Neither experiment produced noteworthy results; more work exploring hyperparameter tuning is required to determine the applicability of this method to our data.

## 3. Results

### 3.1. Cohort 1 Experiment

The Cohort 1 experiment demonstrated a highly promising outcome with the BSP algorithm, as shown in Table 2. While the ensemble run using raw inputs and AHE performed well during testing and on the active set, the snapshot ensemble did not have enough confidence to reach the threshold, suggesting unstable training and thus disagreement between the last three snapshots. Meanwhile, BSP produced good accuracy and excellent confidence, suggesting stability in the last three snapshots. GDBSP’s accuracy appears extremely limited: upon analyzing the data, it appears that this preprocessing method suffers from data starvation when too few samples are included in the training set.

Ultimately, BSP was able to grow the dataset by 66% and increase accuracy by 1.9% on the test folds and 3% on the remaining active set. We can conclude that BSP and active learning improve the effectiveness of an ensemble and its generalizability to unknown data. With this conclusion in mind, we move on to active learning performed on Cohorts 2 and 3.

### 3.2. Cohorts 2 and 3 Experiment

From Table 3, we infer that our ensemble shows the best confidence with GDBSP, improving to 96.5% in the third iteration. The active set sample integration of AHE and BSP is excellent, but that of GDBSP is outstanding. Keep in mind that the base dataset (training/validation/testing) consists of Cohort 1, with 4513 samples. With the raw input images, DoG, and HE, no samples reach the minimum 90% confidence threshold. Histogram matching and BSP are able to grow the dataset to 170% and 168% of its original size, respectively. AHE greatly improves on this, growing the dataset to 272%. GDBSP improves on this further, reaching 286%.

Qualitatively, the expert opinion during the sample review process is that the segmentations for AHE and GDBSP improved markedly over time; in addition, samples that previously were evaluated poorly improved in later iterations to be accepted, especially those from Cohort 3. This will be discussed below.

It is notable that the advanced methods (i.e., AHE, BSP, and GDBSP) appear to have no effect or sometimes decreased Dice accuracy during testing on Cohort 1 over their iterations. Next to the expert opinion that segmentation quality on the active set was improving, this is a puzzling outcome. However, we will present below a working hypothesis for why this might be the case.

An analysis of expert time shows clear wins for automatic labeling. Manual labeling of ROI on these high-resolution images might require days of expert effort but with automatic labeling, we are able to reduce this to a mere handful of hours. In our full evaluation of GDBSP shown in Table 4, we effect a 96% reduction in expert working time, while the dataset size is multiplied by more than a factor of eight.

### 3.3. GDBSP Preprocessing

**Figure 8:**
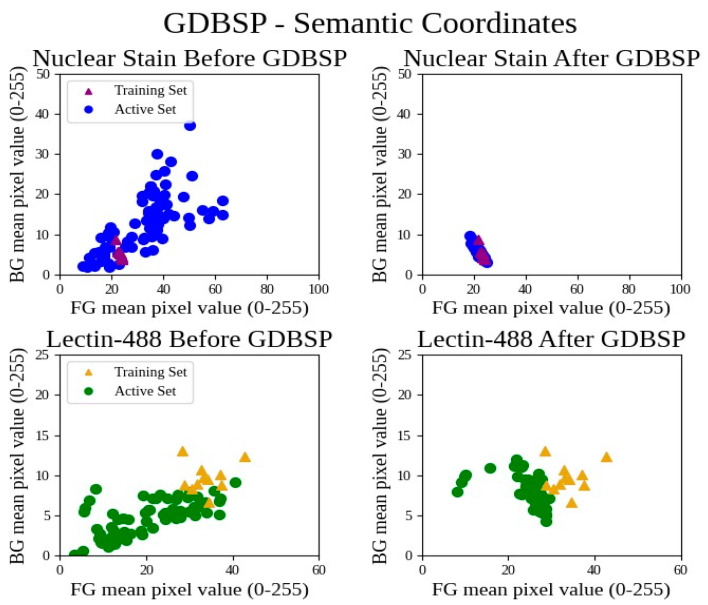
Point clusters demonstrating GDBSP preprocessing in unknown sample normalization.

GDBSP was applied on both nuclear stain and lectin-488 images. The left panels of Figure 8 reveal that the pixel values of both foreground and background in the active set were significantly scattered compared to those in the training set across both image channels (nuclear stain and lectin-488) before image preprocessing. However, after image processing (right panels), the pixel values of both foreground and background in the active set were much closer to those of the training set. As demonstrated in Figure 8, the point clusters indicated how GDBSP preprocessing normalizes the unknown active set to the known training set in the semantic feature space. The axis ranges are zoomed in for best interpretability. GDBSP is able to tightly cluster all of the training set and active set features for the nuclear stain channel; however, the active set (Cohorts 2 and 3) lectin-488 channels cluster proximal to, but with a different mean than, the training set (Cohort 1). This outcome likely explains the drop in Cohort 1 test accuracy, but the improvement in active set label quality.

## 4. Discussion

BSP shows robustness with small initial datasets, while GDBSP demonstrates increased sensitivity or vulnerability when applied to the datasets. However, when there is sufficient data, BSP outperforms all other methods in automatic labeling. This suggests that BSP might serve well as a first step in a data-starved scenario, followed by GDBSP or AHE in subsequent iterations. The raw-input control, DoG, and HE experiments accepted no samples. This outcome is substantially worse than Alahmari et al.’s results [34], suggesting that the nature of the dataset has a large effect on acceptance results: even at a 90% minimum confidence threshold, equivalent to their experiment, no samples reach it.

We can compare the sample acceptance rates of the various preprocessing methods with the original ADL implementation, where Alahmari et al. averaged 11% of the active set per iteration [34]. AHE improves over ADL, at 13.9% of the active set on average per iteration. GDBSP greatly improves over ADL, at 18.1% of the active set per iteration on average, even with much stricter 98%+ confidence thresholds relative to Alahmari et al.’s 90%. Thus, GDBSP provides a large improvement in the automatic labeling capabilities of the ADL method.

The accepted sections for BSP and GDBSP tend to weigh heavily toward Cohort 2 in early iterations, with Cohort 3 only appearing in later iterations if at all. Analysis of Cohort 3 lectin-488 images reveals a possible cause: these were imaged with an additional staining for dystrophin proteins with the use of an Alexa Fluor 594 secondary antibody, which has a slight bleed-through in the lectin-488 imaging wavelength. This additional stain brightens the background and the nonsignal areas of lectin-488 images, as is shown in Supplementary Figure S2.

GDBSP shows strong gains on these very Cohort 3 images deeper in its iterations. By iteration 5, samples that previously produced poor segmentations suddenly were being evaluated as very high quality. Our hypothesis is that this may be due to active deep learning “finding the universal mean”, as the accepted data take over the bulk proportion of the training set. At least we are discovering the mean of our collected data, which occurs even as its test Dice on Cohort 1 drops slightly, though not enough to be statistically significant. This situation is also similar for both AHE and BSP. It could be that, as an extension to our earlier theories on bias and variations from microscopic image acquisition, the small initial cohort simply does not best represent the wider universe of possible data. As we fit the model to more data and discover the new mean, we might expect that our fit quality diminishes on the initial dataset. Thus, the accuracy of the model on Cohort 1 testing is not necessarily reflective of its accuracy in the wider universe of data.

Analysis of the Cohort 1 test segmentations produced by GDBSP in its later iterations supports this hypothesis in that several outlier samples produce particularly poor Dice scores. On reflection, these fall into two categories: (1) samples that would be rejected on the second analysis for manual ground truth and inclusion in the initial dataset due to poor sectioning or imaging quality, and (2) samples for which the manual ground truth was of low initial quality. In the second case, the expert determined that the segmentation produced by GDBSP, which was nominally scored with a low Dice value, would make a more accurate ground truth. Of the 11 Cohort 1 sections, two were targeted as potential candidates to be discarded, and another three were targeted as potential candidates for relabeling.

The model was robust in this scenario, in a qualitative experts’ opinion, and was able to improve its performance on unknown data via the active learning process, especially on Cohort 3 samples. We intend to study this ability for active deep learning to help experts find the mean of their data, characterize their qualities, and flag expert mistakes in our future work.

Besides technological advancement, when working with medical images, ethical considerations must be carefully addressed. Medical image interpretation often involves the use of sensitive medical data, including patient images and health records. It is crucial to ensure that patient privacy is protected throughout the entire process, from data storage to analysis and interpretation. This requires implementing robust security measures. Medical imaging datasets are valuable assets that need to be safeguarded against cyber threats and breaches. Also, automated image interpretation algorithms can be susceptible to biases that may result in inaccurate decision making. Therefore, it is crucial to involve diverse and representative training datasets, algorithmic transparency and accountability, and ongoing monitoring and evaluation of algorithm performance by medical experts.

## 5. Conclusions, Limitations, and Future Work

We have shown that active deep learning for automatic labeling, a promising method for expanding the ground truth of datasets, is sensitive to inconsistent microscopy conditions, and tends to overfit the training data distribution. We hypothesized that for more effective evaluation and automatic creation of ground truths for uncertain samples, they should be normalized to the training set distribution using preprocessing adjustments that adequately cover the space of possible variance of a particular image type. We proposed a semantic preprocessing method that bootstraps semantic features contained in predictions to robustly apply these adjustments, avoid unwanted overfitting, and generate high-quality normalizations and ROI labels from unlabeled data. Compared to Alahmari et al.’s active deep learning method without our preprocessing method, even with a higher confidence threshold, we can greatly increase the rate of sample acceptance. We accept 92% of the automatic labels generated for our unlabeled data cohort, thereby expanding the labeled dataset by 845%. We demonstrate a 96% time savings on data labeling for medical researchers. We demonstrated this performance through patch-wise interpolation of high-resolution whole-slide images, allowing us to fully preserve image detail and region label fidelity with no loss of information due to downscaling.

We observed that increasing segmentation quality on unknown data, especially out-of-distribution samples from an unlabeled cohort, correlated with stagnant or decreasing Dice scores on the initial cohort. This suggests that the accuracy of the initial cohort is not reflective of the accuracy of the wider universe of data. We hypothesize that active learning and automatic labeling allow us to discover the universal data mean, or at the least, the mean of our total data pool. We gathered evidence for this hypothesis by observing five outlier images from Cohort 1 that the expert identified as ‘poor-quality’, with the ground truth labels potentially in need of regeneration.

To effectively adopt the approach presented in this work, two key factors must be considered. Firstly, high-quality images with expert ground truth annotations are essential. Secondly, it is crucial to understand the key features contributing to ‘low-quality’ images and to identify or develop appropriate preprocessing algorithms to be incorporated into the pipeline. These measures streamline the process and enhance the effectiveness of the approach. As exemplified in this work, inconsistencies in the dataset manifest as variable levels of image brightness and contrast. By selecting images with ideal brightness and contrast levels, along with an expert ground truth segmentation map, we establish target brightness levels for both the foreground and background. Subsequently, we calculate the Euclidean distance between the brightness of the foreground and background in the image being evaluated and the target image with ideal brightness and contrast for generating ground truth from the active dataset.

Our next steps involve exploring the types of semantic preprocessing possible to integrate as an iterative bootstrapping solution, and new datasets to use it with. We believe that other types of datasets beyond histopathological images could also be handled this way, such as diagnostic magnetic resonance images (MRI). In this case, metrics such as Fourier k-space data could assist adjustment. Having established the value of semantic preprocessing, it should be possible to design more complex and powerful algorithms to advance active learning and automatic labeling methods for myriad applications. Formulating a method for measuring accuracy beyond the initial cohort would help us measure generalization on unknown data. Measuring the adaptability of active learning over its iterations may also prove fruitful, possibly by providing a model with data known to be outliers as the initial dataset. We might also reformulate our quality metric to reflect a confidence measurement. Extracting the per-pixel confidence vote values and using them to construct a confidence map of each sample would give us a quality metric similar to previous types of active learning and help the experts determine where to focus their attention.

## Figures and Tables

**Table 1:** Dataset sample counts.

Data Subset	Number of Samples
Cohort 1	4513 (with ground truth)
Cohort 2	10,604
Cohort 3	10,684
Active set total (cohorts 2 + 3)	21,288
Total	25,801

**Table 2:** Cohort 1 experiment results.

Preprocessing	Iteration	Mean 6-Fold Test Dice	Active Set Mean Dice	Active Set Mean Confidence	97% Conf Threshold Samples	Accepted Samples	Dataset Size after Acceptance
Raw	1	87.8%	89.4%	76.7%	0	0	100%
DoG	1	40.9%	40.7%	68.3%	0	0	100%
HE	1	83.9%	78.9%	86.7%	0	0	100%
AHE	1	90.1%	94.6%	92.2%	0	0	100%
HM	1	79.4%	79.0%	65.9%	0	0	100%
	1	91.8%	91.5%	96.7%	777	399	116%
BSP	2	93.5%	92.2%	97.7%	1618	778	147%
	3	93.7%	94.5%	97.4%	840	462	166%
GDBSP	1	82.2%	53.0%	92.4%	0	0	100%

**Table 3:** Cohorts 2 and 3 Experiment—Sample Acceptance Results.

Preprocessing	Iteration	Mean Active Conf.	Chosen Threshold	% Samples Accepted	Cum. Accepted Samples	Cum. Expert Time (h)	Cum. Manual Expert Time (h)	Dataset Size
Raw	1	80.4%	90.0%	0%	0	0.0		100%
DoG	1	63.5%	90.0%		0	0.0		100%
HE	1	83.4%	90.0%		0	0.0		100%
	1	89.1%	93.0%	69%	1411	0.6	14	131%
AHE	2	94.1%	97.5%	100%	4881	1.7	40	208%
	3	93.9%	97.7%	100%	7767	2.6	62	272%
	1	95.2%	97.5%	48%	831	0.5	10	118%
HM	2	92.7%	96.0%	55%	2271	1.1	22	150%
	3	93.3%	96.5%	47%	3171	1.5	30	170%
	1	92.3%	96.0%	43%	584	0.5	10	113%
BSP	2	96.0%	98.0%	49%	1861	1.1	22	141%
	3	95.5%	98.0%	44%	3060	1.6	32	168%
	1	96.1%	98.0%	92%	2748	0.9	22	161%
GDBSP	2	96.6%	98.5%	93%	5804	1.9	44	229%
	3	96.5%	98.3%	90%	8374	2.7	64	286%

**Table 4:** GDBSP Full Run Results.

Iterations	Cumulative Accepted Sections	% Active Set Accepted	Dataset Size	Cumulative Expert Time (h)	Equivalent Manual Expert Time (h)	Expert Time Saved
10	82	92%	845%	7.2	164	96%

## Data Availability

All the data is included in this article.

## Supplementary Materials

The following supporting information can be downloaded at: https://www.mdpi.com/article/10.3390/bioengineering11050434/s1, Figure S1: Demonstration of an overfitting ensemble misclassifying labels on a Cohort 3 section, lectin-488 channel. Left is the “adjusted” image, which was unchanged by SP from the raw image; Figure S2: Dice accuracies of iterative preprocessing using distance-minimization BSP; Figure S3: Average brightness for the foreground and background regions of the nuclear stain and lectin-488 channels of the dataset. Discussion S1: BSP Adjustment results; Discussion S2: Dataset Inconsistency.
