# Supplementary material for: Synergizing Deep Learning-Enabled Preprocessing and Human–AI Integration for Efficient Automatic Ground Truth Generation
Source: Bioengineering (Basel). 2024 Apr 28;11(5):434. doi: 10.3390/bioengineering11050434 (PMC11117745; doi:10.3390/bioengineering11050434)
Supplement: Supplementary file 1 [file bioengineering-11-00434-s001.zip › bioengineering-2963122-supplementary.pdf]

Synergizing Deep Learning-Enabled Pre-processing and Human-AI Integration for Efficient Automatic Ground Truth Generation

Christopher Collazo[1], Ian Vargas[2], Brendon Cara[2], Carla J. Weinheimer[3], Ryan Grabau[2], Dmitry Goldgof[1*], Lawrence Hall[1*], Samuel Wickline[2], Hua Pan[3,4,5*]

[1]University of South Florida College of Engineering

[2]University of South Florida College of Medicine

[3]Washington University in St. Louis Department of Medicine

[4]Washington University in St. Louis Department of Pathology & Immunology

[5]Washington University in St. Louis Department of Biomedical Engineering

* Correspondences: E-mail: goldgof@usf.edu, lohall@usf.edu, and hpan@wustl.edu

Supplementary Material

Supplementary Figure

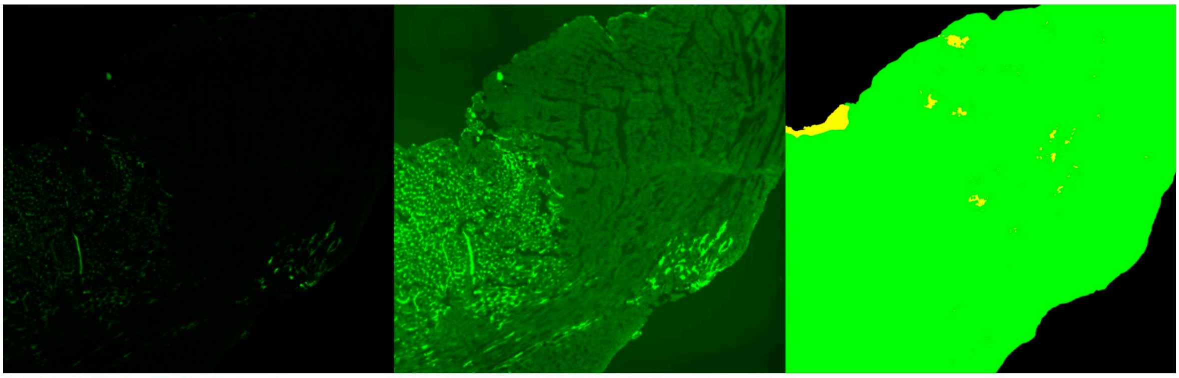

False-color raw image   Exposure-adjusted image   Ensemble output

Supplementary Figure S1. Demonstration of an overfitting ensemble misclassifying labels on a Cohort 3 section, lectin-488 channel. Left is the “adjusted” image, which was unchanged by SP from the raw image. Middle is the same, exposure boosted for visibility. Right is the ensemble voted segmentation map. Here we see significant differences in non-signal region background noise which are not present in Cohort 2 or the original training set, and see that the model misclassifies these regions as ”normal”.

Supplementary Discussion

I. Supplementary Discussion S1 - BSP Adjustment results

As covered in Bootstrapped Semantic Preprocessing (BSP), starting from an approximation, we apply SP and predict iteratively, asymptotically approaching an accurate segmentation. A reasonable number of iterations can be chosen which balances a tradeoff between accuracy and evaluation speed. We chose 5 iterations, with the Otsu preliminary map acting as a

0th iteration. In Supplementary Figure S2, we see that while the accuracies of the initial Otsu threshold maps are low, the follow-up SP-prediction iterations improve accuracy each time. Most convergence finishes by iteration two, with minor improvements beyond that point.

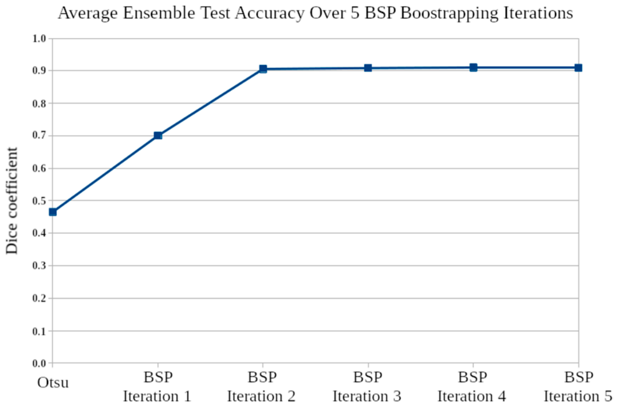

Supplementary Figure S2. Dice accuracies of iterative preprocessing using distance-minimization BSP. Observe that most convergence finishes by iteration two, with minor improvements beyond that point.

II. Supplementary Discussion S2 – Dataset Inconsistency

To demonstrate the whole-slide data collection inconsistency problems semantic preprocessing (SP) works towards solving, we use a derived semantic metric in this experiment. This is discussed in greater depth in the Semantic Preprocessing section of the main paper. In summary, the two input channels are each separated into a binary “signal” foreground and “nonsignal” background according to the ground truth segmentation map, becoming binary images.

The Cohort 1 sections manifest interesting and sometimes extreme variations in brightness from the Cohort 1 average. The most obvious example is Process 277, or p277. In Supplementary Figure S3, we see the lectin-488 channel exhibits an exceedingly high foreground average pixel value, with a likewise elevated background average pixel value. As well, p272’s lectin-488 channel averages lie within a constricted range.

Several algorithms tested in the described experiments require an adjustment target. The expert-selected target for all such tested algorithms is the image properties of section p177. Its average intensity levels represent a high-quality signal separation between foreground and background, as well as a close match to the dataset average. Further, p177 consistently receives high-accuracy evaluations from models where it is left out as the “test” section, suggesting it is a suitable representative of the dataset average.

Similar variances to those in the training set are present in the active set, shown in Figure S2 of the main paper. Cohort 2’s nuclear stain channel is exceptionally bright, with most of its interquartile range corresponding to Cohort 1’s upper quartile. Cohort 3’s lectin-488 channel skews bright, with its upper quartile matching Cohort 1’s maximum. This suggests, and a manual analysis confirms, that the Cohort 3 lectin-488 backgrounds are much brighter, exhibiting a kind of overexposure. These differences are exactly the sort of inconsistency in microscopy that SP is meant to handle.

https://github.com/nervecenter/bsp-experiment

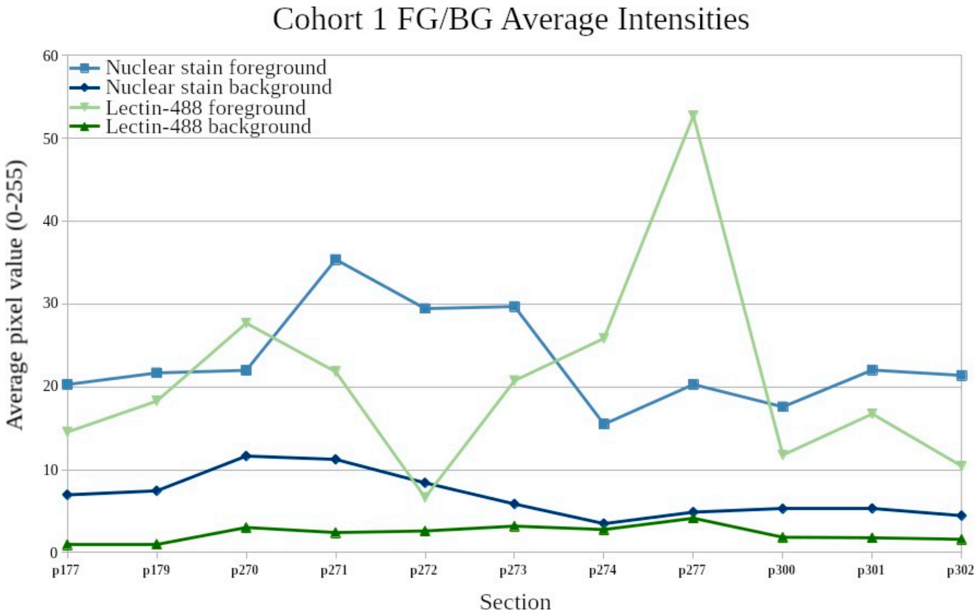

Supplementary Figure S3. Average brightness for the foreground and background regions of the nuclear stain and lectin-488 channels of the dataset. The two channels are adjusted independently. Note that p177 is the target. Also note p277's large green-channel deviation.

https://github.com/nervecenter/bsp-experiment
